# Impact of the Omicron Strain on Febrile Convulsions Requiring Hospitalization in Children: A Single-Center Observational Study

**DOI:** 10.3390/pediatric16020034

**Published:** 2024-05-14

**Authors:** Masayuki Nagasawa, Teruyoshi Shimoyama, Sayuri Hashimoto, Ryuichi Nakagawa, Haruna Yokoyama, Mari Okada, Tomohiro Udagawa, Akihiro Oshiba

**Affiliations:** Department of Pediatrics, Musashino Red Cross Hospital, 1-26-1, Kyonan-cho, Musashino 180-8610, Tokyo, Japan; terustarrysky@gmail.com (T.S.); sayuri.hashimo@gmail.com (S.H.); uda0112@yahoo.co.jp (T.U.); aoshiba@musashino.jrc.or.jp (A.O.)

**Keywords:** febrile convulsion, respiratory virus, multiplex PCR, COVID-19, SARS-CoV-2, Omicron strain

## Abstract

Background. The emergence of the Omicron strain of severe acute respiratory syndrome coronavirus 2 (SARS-CoV-2) at the end of December 2021 has drastically increased the number of infected children in Japan, along with the number of children with febrile convulsions, but its clinical impact is unclear. Materials and Methods. We compared the frequency of SARS-CoV-2 infection in children hospitalized with febrile convulsions with the frequency of SARS-CoV-2 infection in children with fever and respiratory symptoms without convulsions. Results. In 2021 and 2022, 49 and 58 children required emergency hospitalization for febrile convulsions (FC group) with status epilepticus or cluster spasms, in which 24 and 38 children underwent a Filmarray^®^ respiratory panel test (FA test), respectively, and others received a quantitative antigen test for SARS-CoV-2. In 2022, only six patients tested positive for SARS-CoV-2 (10.3%, 6/58). As a reference group, 655 children aged <10 years who underwent the FA test for fever and respiratory symptoms during the same period were investigated, and 4 (1.8%, 4/223) and 42 (9.7%, 42/432) tested positive for SARS-CoV-2 in 2021 and 2022, respectively. Rhinovirus/enterovirus (RV/EV) was the most frequently detected virus (40.3%, 264/655), followed by respiratory syncytial virus (RSV) (18.9%, 124/655) and parainfluenza virus 3 (PIV3) (7.8%, 51/655). There was no significant difference in the trend of detected viruses between the two groups. Conclusions. The frequency and severity of febrile convulsions requiring hospitalization associated with SARS-CoV-2 infection of the Omicron strain may be similar to that of other respiratory viruses in children.

## 1. Introduction

**Figure 1:**
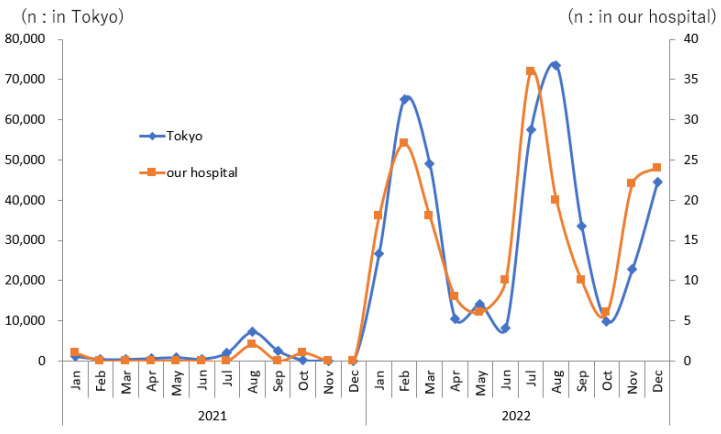
Trend of severe acute respiratory syndrome coronavirus 2-infected patient number under 10 years old in Tokyo [18] and our hospital.

The coronavirus disease 2019 (COVID-19) pandemic caused by severe acute respiratory syndrome coronavirus 2 (SARS-CoV-2) has had a significant impact worldwide [1,2]. In Japan, the pandemic has greatly influenced socio-economic activities. Moreover, a strong impact on trends in acute epidemics other than COVID-19 was also observed, with a decline in invasive pneumococcal infections and the disappearance of seasonal influenza epidemics [3,4,5]. Japan had experienced eight waves of COVID-19 epidemics by March 2023, and the number of infected children had increased drastically, especially after the sixth wave caused by the Omicron strain, which began at the end of December 2021. As presented in Figure 1, the number of infected children aged <10 years in Tokyo increased by 26-fold in 2022 compared to that in 2021, while the number for all ages increased by 11-fold in 2022 compared to that in 2021. Similar to reports from overseas [6,7], the symptoms of pediatric patients with COVID-19 in Japan were generally milder than those of adults; however, with the rapid increase in the number of pediatric patients, a certain number of severe cases and even deaths have been reported among them [8,9,10]. In pediatric emergency care facilities, a surge in pediatric patients with COVID-19-related convulsions has been observed, and cases of encephalopathy have also been recorded [11,12]. Convulsions associated with acute viral illnesses are a common neurological complication in children [13,14,15]. Previous studies have reported that the recently introduced multiplex polymerase chain reaction (PCR) method can simultaneously analyze multiple types of respiratory viruses and detect viruses at a higher rate than conventional methods [16,17]. However, few reports to date have compared the frequency of COVID-19-related convulsions with that of other viral diseases. Hence, we examined the results of the Filmarray^®^ respiratory panel (FA test; ver. 2.1, BioMerieux Japan, Tokyo, Japan), a multiplex PCR test, and a quantitative antigen assay for SARS-CoV-2 in children hospitalized with febrile convulsions and compared them to those in whom the FA test was performed for respiratory symptoms in 2021–2022 at our institution. Our observational study has shown that the frequency of febrile convulsions requiring hospitalization associated with the Omicron strain of SARS-CoV-2 may not be higher and its severity even less than that associated with other common respiratory viruses.

## 2. Materials and Methods

### 2.1. Patients

Our institution is a tertiary emergency general care hospital located in the Tokyo metropolitan area, adjacent to western central Tokyo.

Patients aged <10 years who were admitted urgently to our hospital because of status epilepticus or cluster spasms associated with fever (≥38 °C) between January 2021 and December 2022 were included in this study as the febrile convulsion group (FC group). Patients diagnosed with epilepsy and those taking antiepileptic medication were excluded. Meanwhile, patients aged <10 years with fever and/or respiratory symptoms in which the FA test was deemed necessary by the physicians during the same period were included as the reference group. Most cases who received the FA test required hospitalization. Clinical information such as patient age, hospitalization period, clinical symptoms, and blood data was collected as anonymized information from electrical medical records.

### 2.2. Public Survey of Infectious Disease Outbreak Trend in Tokyo

The Infectious Disease Outbreak Trend Survey is a research project that has been conducted nationwide since 1981 in Japan. Infectious Disease Outbreak Trend Surveys monitor two types of infectious diseases: “infectious diseases subject to complete monitoring”, which require the notification of all cases, and “infectious diseases subject to sentinel monitoring”, which require reports of patients diagnosed at designated reporting institutions (sentinel medical institutions). Infectious diseases that require complete identification are those that occur in rare numbers or that require measures to prevent the spread of infection to surrounding areas. Infectious diseases that are monitored at fixed points are those whose incidence trends need to be ascertained, but where the number of patients is large and it is not necessary to track all cases. Tokyo has 264 pediatric sentinel medical institutions and 155 internal medicine sentinel medical institutions for this survey.

COVID-19 had been monitored as “infectious diseases subject to complete monitoring” until May 2023, and has been monitored as “infectious diseases subject to sentinel monitoring” thereafter in Japan.

The results of the infectious disease trend survey are updated weekly and made available to the public on the web (https://idsc.tmiph.metro.tokyo.lg.jp/weekly/, accessed on 19 April 2023).

### 2.3. Multiplex PCR Test

The FA test (BioMerieux Japan, Tokyo, Japan) was used to detect respiratory viruses from nasopharyngeal swab samples from patients at the discretion of the physicians. The FA test is a method that uses multiplex nested PCR to detect multiple pathogens at once using an automatic analyzer. The panel test can detect 18 viruses as follows: adenovirus (AdV); coronaviruses HKU1, 229E, OC43, and NL63; SARS-CoV-2; influenza A, A/H1, A/H1 2009, A/H3, and B; parainfluenza virus (PIV)-1, -2, -3, and -4; respiratory syncytial virus (RSV); rhinovirus/enterovirus (RV/EV); human metapneumovirus (hMPV); and four other microorganisms including *Bordetella pertussis*, *Bordetella parapertussis*, *Chlamydia pneumonia*, and *Mycoplasma pneumoniae*. Point-of-care antigen detection tests for influenza A/B (flu A/B), AdV, RSV, and hMPV were applied based on the physician’s discretion for patients who did not undergo an FA test.

### 2.4. Quantitative Antigen Assay for SARS-CoV-2

For all the hospitalized patients who did not undergo an FA test, a SARS-CoV-2 quantitative antigen test was performed at admission as part of the infection control strategy of our hospital. The HISCL™ SARS-CoV-2 Ag kit (Sysmex, Kobe, Japan) was used for a quantitative assay for the SARS-CoV-2 antigen [19] until July 2022 and has been replaced with the Elecsys^®^ SARS-CoV-2 antigen kit (Roche Diagnostics Japan, Tokyo, Japan) since August 2022. According to an in-house validation study, the two assays had no significant differences in terms of sensitivity and specificity for detecting the SARS-CoV-2 antigen.

### 2.5. Statistical Analysis

Fisher’s exact test and Wilcoxon rank sum test were performed for statistical analysis, and *p* < 0.05 was considered significant. Statistical analyses were carried out using JMP 14 (SAS Institute, Cary, NC, USA).

## 3. Results

In 2021 and 2022, 49 and 58 children, respectively, required emergency hospitalization for febrile convulsions (designated as the febrile convulsions [FC] group). Their average ages were 31.5 ± 20.7 months (5–112 months old) and 35.9 ± 29.8 months (7–165 months old), respectively. No significant difference in the length of the average hospital stay was observed between 2021 and 2022 (4.17 ± 4.17 days vs. 4.35 ± 2.35 days) (Table 1).

In 2021, 24 of the 49 patients underwent the FA tests, and the virus was detected in 18 patients (75.0%). None of the patients tested positive for SARS-CoV-2 using the FA or quantitative antigen tests. Seven other patients underwent the AdV antigen test, and three underwent the RSV antigen test, all of whom tested negative. In 2022, 38 of 58 patients underwent the FA tests, and the virus was detected in 24 patients (63.2%). Additionally, one AdV antigen test and one RSV antigen test were performed, both of which yielded negative results. The SARS-CoV-2 antigen quantitative test was performed in all the patients who did not undergo the FA test, and four patients tested positive in the quantitative antigen test in 2022 only. In 2022, two patients tested positive for SARS-CoV-2 in the FA test. By contrast, 655 children aged <10 years (average age: 34.7 ± 27.5 months, 0–4 years old: 536 patients, and 5–9 years: 119 patients) with fever and respiratory symptoms during the same period underwent the FA test (designated as the reference group), and 4 and 42 children were positive for SARS-CoV-2 in 2021 and 2022, respectively. One or more viruses were detected in 74.0% (165/223) and 69.7% (301/432) of the patients, respectively. There was no significant difference in average age between the FC group and reference group (33.7 ± 28.5 vs. 34.7 ± 27.5 months, *p* = 0.897).

**Figure 2:**
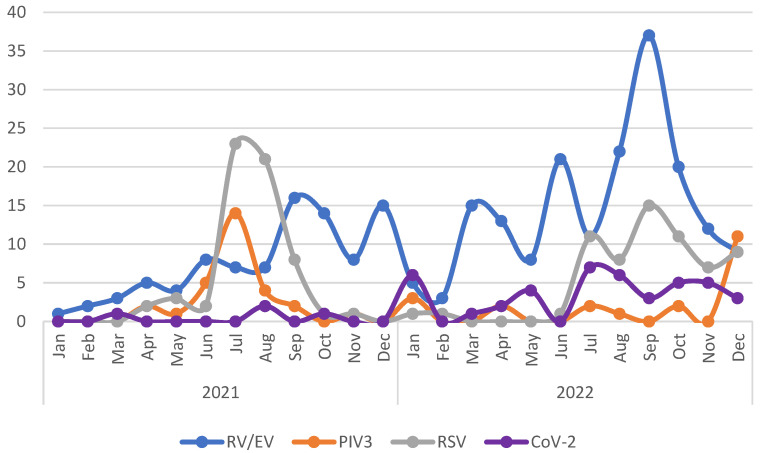
Trend of four major respiratory viruses detected by Filmarrray^®^ respiratory panel (ver2.1) test in children under 10 years old in the reference group. RV/EV: rhinovirus/enterovirus, PIV3: parainfluenza virus 3, RSV: respiratory syncytial virus, CoV-2: severe acute respiratory syndrome coronavirus-2.

The trends in the number of pediatric patients with COVID-19 aged <10 years officially registered in Tokyo and outpatients aged <10 years who visited our hospital with mild respiratory symptoms or fever and tested positive for quantitative antigen assay are shown in Figure 1. The epidemic trend of infection was identical. The trends of detected viruses in the reference group are presented in Figure 2. There is a seasonal trend in RSV and PIV3 infections. On the other hand, RV/EV infections are generally prevalent throughout the year, although there are some fluctuations. These observations are similar to the previous reports from overseas and Japan [20,21]. RV/EV was the most frequently detected virus in both groups, followed by RSV and PIV3 (Table 2). Multiple viruses were detected in 12.9% (8/62) of the patients in the FC group and in 15.1% (99/655) of the patients in the reference group, which was not significantly different. Biphasic encephalopathy was reported in a patient who tested positive for PIV3 in 2021, and in another patient who tested positive for PIV1 and RV/EV in 2022. No significant difference in the frequency of viruses detected was observed between the FC and reference groups in 2021 and 2022 (Table 2).

Table 3 presents the age, length of hospitalization, type of convulsion, and frequency of first FC episode in patients positive and negative for the FA test, as well as positive for each virus. There was a tendency for the FA-test-positive cases to be slightly younger, but there was no significant difference (*p* = 0.17). There was no significant difference between the length of hospitalization and frequency of first FC episode between RV/EV, RSV, PIV3, and SARS-CoV-2 infections. The frequency of status epilepticus in RV/EV infection was significantly higher than that in SARS-CoV-2 infection (odds ratio 95%CI: 1.7–112.6, *p* < 0.05).

## 4. Discussion

Febrile convulsions are seizures that can occur in children as a result of fever [22]. Febrile convulsions are usually classified as simple or complex. Simple febrile convulsions are the most common type that lasts from a few seconds to 15 min. Simple febrile convulsions do not recur within a 24 h period and usually are not specific to one part of the body. On the other hand, complex febrile convulsions are a type that lasts longer than 15 min, occurs more than once within 24 h, or is confined to one side of your child’s body. Status epilepticus, a serious form of complex febrile convulsions, is defined as seizures lasting more than 30 min or recurrent seizures with no recovery of consciousness between attacks for more than 30 min [13,23]. It has been reported that simple febrile convulsions are not associated with any permanent neurological deficits [24]. Young children between the ages of about 6 months and 5 years old are the most likely to have febrile convulsions. They are relatively common, affecting around 3% of all children, and it has been reported that about 2% to 5% of children in the US and Western Europe, 6% to 9% of infants and children in Japan, 5% to 10% in India, and as high as 14% in Guam will have experienced at least one febrile seizure by the age of 5 years, indicating some ethnic differences [23]. There are no specific data available for simple febrile convulsions.

In the case of status epilepticus or complex febrile convulsions, it may be an early symptom of bacteremia, meningitis, or encephalitis/encephalopathy. Hospitalization observation is generally recommended as well as imaging examination, blood tests, and cerebrospinal fluid tests, if required. It has been reported that the risk of developing epilepsy is increased further in children with a history of complex febrile convulsions [25,26]. It has also been reported that a strong association exists between febrile status epilepticus or febrile convulsions characterized by focal symptoms and the later development of temporal epilepsy [27,28].

Febrile convulsions can be caused by a variety of viral and bacterial infections, including respiratory viruses such as influenza, respiratory syncytial virus (RSV), human metapneumovirus (hMPV). It has been reported that about 30 to 40 percent of children who have one febrile seizure will have another. Children whose family members had febrile seizures are more likely to have more than one seizure [13].

There are already several reports that the introduction of multiplex PCR testing has increased the virus detection rate significantly in patients with febrile convulsions.

In a report from Germany [17], the detection rate of respiratory viruses in children with febrile convulsions was 10% (10/49) before the introduction of multiplex PCR testing, but this increased to 71.2% (52/73) after the introduction. The multiplex virological panel (FTD Respiratory pathogens 21, Fast Track Diagnostics, Luxembourg) used in this study included the following viruses: influenza A/H1N1, influenza B, parainfluenza type 1, 2, 3, 4, coronavirus (NL63, 229E, OC43, HKU1), human metapneumovirus, human bocavirus, rhinovirus, adenovirus, respiratory syncytial virus (RSV A/B), parechovirus, and enterovirus. The most commonly detected viruses by multiplex PCR testing were adenovirus (*n* = 12), human bocavirus (*n* = 10), enterovirus (*n* = 9), rhinovirus (*n* = 7), RSV (*n* = 7), human coronavirus (*n* = 7), parechovirus (*n* = 5), parainfluenza virus (*n* = 5), and human metapneumovirus (*n* = 3). More than one viral pathogen was detected in 16 of 52 cases (30.7%). Of note, simple febrile convulsions accounted for 64.5% in this study.

A report from South Korea [16] states that 235 children hospitalized with febrile convulsions were subjected to multiplex PCR testing, and 112 were positive for respiratory viruses (81.2%). The commercial multiplex PCR test kit used (Anyplex™ II RV16 Detection kit (Seegene Inc., Seoul, Republic of Korea)) can simultaneously identify 16 types of respiratory virus, including adenovirus, influenza A virus, influenza B virus, parainfluenza virus (types 1, 2, 3, and 4), rhinovirus, bocavirus, coronavirus (229E, OC43, and NL63), enterovirus, human metapneumovirus (hMPV), and RSV (groups A and B). Rhinovirus (*n* = 64) was most frequently identified by the multiplex PCR test, followed by adenovirus (*n* = 31) and enterovirus (*n* = 26). A total of 55 of these (49.1%) were positive for two or more viruses. Of note, simple febrile convulsions in this study accounted for 62.7%. It was discussed that the discrepancy in frequently identified viruses in children with febrile convulsions among studies seems to not be caused by the difference in neuro-tropism of a specific respiratory virus, but to be caused by the epidemic extent of each virus during the study period.

In our institute, the FA test was introduced in January 2021. The FA test has been able to detect the respiratory viruses in as much as 70% to 80% of the children under 5 years of age who were admitted to our department because of fever and/or respiratory symptoms at our institute [29], which is similar to other reports [30,31,32]. Before 2021, only the point-of-care antigen test for influenza A or B, respiratory syncytial virus, human metapneumovirus, and adenovirus were available to detect respiratory viruses for clinical use at our institute.

When we retrospectively examined 512 children under the age of 10 years who were admitted to our department because of status epilepticus or cluster spasms during fever from 2012 to 2020, at least one point-of-care antigen test described above was applied in 343 patients, and respiratory viruses were detected in 98 patients (28.6%) (Supplementary Table S1, unpublished data). The influenza point-of-care antigen test was positive in 62 patients, that for the RSV antigen was positive in 20 patients, that for the hMPV antigen was positive in 9 patients, and that for the AdV antigen was positive in 7 patients. No duplicate positive cases were found. The average age of 512 patients was 35.18 ± 31.18 months old, which was not significantly different from that of the FC group in the present study (Table 1). This shows that the introduction of the FA test increased the virus detection rate more than two-fold compared to before (67.7% (42/62) vs. 28.6% (98/343)). Multiple viruses were detected in 12.9% (8/62) of the patients in the FC group and in 15.1% (99/655) of the patients in the reference group in our study. It is unclear why there were fewer patients with multiple viruses detected in our study than in reports from Germany [17] and South Korea [16]. In the two studies from Germany and Korea, approximately two-thirds of the cases were simple febrile convulsions, whereas our cases were all complex febrile convulsions. During our research period of 2021 to 2022, there was no influenza epidemic, due to the effects of the coronavirus pandemic [3,4,5], and there were no patients with febrile convulsions, due to influenza infection. Considering that 62 out of 98 patients (63.3%) were positive for the influenza point-of-care antigen test in patients hospitalized for status epilepticus or cluster spasms between 2012 and 2020, it is safe to assume that the FA test can detect respiratory viruses in patients who were hospitalized for status epilepticus or cluster spasms much more efficiently. In the cohort of 2021 to 2022, only 12 out of the 62 positive cases were positive for RSV, hMPV, and AdV that could be detected by previous point-of-care antigen tests, which indicates that the virus detection rate was possibly increased by five-fold compared to before the introduction of the FA test.

In 512 patients with febrile convulsions admitted between 2012 and 2020 at our institute, there were 16 cases of encephalitis/encephalopathy (3%), 5 cases of influenza positive, 3 cases of exanthema subitum, 1 case of RSV-positive, and 7 cases of unknown causative virus. In our study of 2021 and 2022, there were two cases of encephalitis/encephalopathy (1.9%), one of PIV3-positive, and another of PIV1- and RV/EV-positive. Considering that there was no influenza epidemic in 2021 and 2022, if influenza-related cases were excluded from the 2012 to 2020 cohort, the frequency of encephalitis/encephalopathy would be 2.4% (11/450), which is almost the same as that in 2021 and 2022. In Japan, influenza-related encephalitis/encephalopathy is the most common, followed by exanthema subitum (HHV-6/7) [33].

As mentioned earlier, simple febrile convulsions are a very common disease, and detailed epidemiological research on them is difficult to carry out. Research to identify and determine the frequency of respiratory viral infections, which are the main causes of fever in febrile convulsions, is even more difficult. Most infants with fever subside within 1–2 days, so they do not necessarily seek medical attention, even if they have simple febrile convulsions. There has been a study based on a medical-record database in the US where the frequency of febrile convulsions in COVID-19 patients was 0.5% (44/8854) and accounted for 1.1% (44/3902) of patients with febrile convulsions aged below 5 years old [34]. Among 44 patients with COVID-19 and febrile convulsions, 30 had simple febrile convulsions, 14 had complex febrile convulsions, and 11 required hospitalization. We have to be careful in interpreting this result, because this study targeted medical records from 1 March 2020 to 19 April 2021, and the analyzed data were before the emergence of the Omicron strain of SARS-CoV-2 worldwide. This study concluded that febrile convulsions may be a less severe neurologic manifestation of COVID-19.

There has been a report examining the clinical symptoms of COVID-19 in children under 18 years of age that convulsive symptoms significantly increased after the emergence of the Omicron strain [11]. Another study has reported that the proportions of young children and patients with complex comorbidities were higher during the Omicron period in Korea, and the proportions of patients with seizures increased significantly compared with the delta period (13.2% vs. 2.8%) [35]. However, these findings do not necessarily mean that the Omicron strain is more likely to cause severe fever-related convulsions than other respiratory viruses. This is possibly thought to be the result of the rapid spread of infection among infants and children who are vulnerable to febrile convulsions after the emergence of the Omicron strain. Most of the febrile convulsions that have suddenly increased are considered to be the simple type, which do not require hospitalization. Neurological manifestations of COVID-19 in childhood have been described but are usually less severe than at other ages [36].

COVID-19 is also known to be related to pediatric acute-onset neuropsychiatric syndrome (PANS), which is characterized by a sudden development of neurological and psychiatric symptoms following infections [37,38,39]. Cases of developing Multisystem Inflammatory Syndrome in Children (MIS-C) after contracting COVID-19 have been reported from Europe and America [40], but at our institution, MIS-C has not been observed, and the number of Kawasaki disease (KD) patients which mimic MIS-C has decreased by two-thirds [41]. These COVID-19-related issues are considered to be questions that need to be further studied and analyzed in the future.

What is new and novel about our study is that we compared the virus detection rate with that of patients of the same age who were hospitalized for acute respiratory infections during the same period and by using the same multiplex PCR test.

As shown in Figure 1, the trends in the number of COVID-19 infections based on all-case monitoring data in Tokyo and the trends in mild COVID-19 infections diagnosed at our hospital were extremely similar. The trends in RSV and AdV detected in pediatric inpatients from 2021 to 2022 at our institute closely reflected the trend of public data monitoring infection trends in Tokyo. For reference, respiratory viruses that are being investigated include RSV, influenza, SARS-CoV-2, adenovirus, human parbovirus-19 (HB-19), and herpes virus 6/7 (HHV-6/7) in the Tokyo metropolitan infectious disease trend survey. Among these, the diagnosis of HB-19 and HHV-6/7 infections is based on clinical manifestations. This indicates that the frequency of virus detection in pediatric patients in which the FA test was carried out at our institution reflects the prevalence of the virus in the surrounding area. Based on these observations, we think that it is safe to assume that the trends of respiratory viruses detected in our institute reflect the respiratory virus infection epidemiology in the designated area, and the analysis method in our study is valid.

This study had some limitations. First, it focused on patients with relatively severe symptoms who visited an emergency center, whereas pediatric patients with mild symptoms were not included in the FA test targets. Second, this was a single-center study with a small sample. Lastly, the indications for FA testing were determined by the physician. In general, FA testing tends to be applied to severely ill patients in the reference group, and patients without severe symptoms are subjected to quantitative antigen testing for SARS-CoV-2.

Quantitative antigen testing for SARS-CoV-2 was performed in outpatients with mild respiratory symptoms and fever and in inpatients who had not undergone FA testing at admission. The proportion of SARS-CoV-2-antigen-test-positive outpatients aged <10 years was 19.1% (205/1701) in 2022 at our hospital (Figure 1), which was significantly higher (*p* < 0.001) than that of patients who underwent FA testing in the same period in this study. According to the official monitoring data available in Tokyo [42], the average positive ratio of PCR or antigen testing for SARS-CoV-2 in 2022 in all generations was 32.3%, and this total number of positive cases accounted for 60.3% (2,181,707/3,615,475) of the officially registered patient number. Epidemiological data demonstrate that children aged <10 years in Tokyo account for 7.4% of the total population, and the number of patients of the same age infected with SARS-CoV-2 accounts for 12.1% of all registered patients in Tokyo in 2022, indicating that the actual number of children infected with SARS-CoV-2 among febrile children could be higher than that observed in our institution [18].

In conclusion, SARS-CoV-2 infection may not be more likely to significantly induce febrile convulsions requiring hospitalization than other common respiratory viral infections in our study. Further, the frequency and severity of febrile convulsions associated with the Omicron strain of SARS-CoV-2 infection in children may be almost the same or even slightly less severe than that of RV/EV, RSV, PIV3, and common respiratory viruses other than influenza virus.

## Figures and Tables

**Table 1:** Comparison of the age and length of hospitalization in febrile convulsions group between 2021 and 2022.

	2021	2022	
patient (n)	49	58	
average age (months)	31.5 ± 20.7	35.9 ± 29.8	*p* = 0.927 *
median age (months)	25.0	26.5	
max (months)	112	165	
min (months)	5	7	
admission period			
average period (days)	4.2 ± 1.4	4.4 ± 2.4	*p* = 0.612 *
median (days)	4	4	
max (days)	8	14	
min (days)	2	2	

* Wilcoxon rank sum test.

**Table 2:** Comparison of each virus detection ratio between the febrile convulsions (FC) and reference groups in 2021 and 2022.

	2021					2022				
	FC		Reference			FC		Reference		
n	49(24)		223			58(38)		432		
RV/EV	33.3%	(8/24)	36.8%	(82/223)	*p = 0.819*	42.1%	(16/38)	42.1%	(182/432)	*p = 0.972*
RSV	22.2%	(6/27)	26.9%	(60/223)	*p = 0.686*	5.1%	(2/39)	14.8%	(64/432)	*p = 0.168*
hMPV	0.0%	(0/24)	0.0%	(0//223)	*n.a.*	7.9%	(3/38)	5.8%	(25/432)	*p = 0.564*
PIV3	25.0%	(6/24)	13.5%	(30/223)	*p = 0.206*	2.6%	(1/38)	4.9%	(21/432)	*p = 0.585*
AdV	3.2%	(1/31)	3.6%	(8/223)	*p = 0.921*	2.6%	(1/39)	6.0%	(26/432)	*p = 0.444*
NL63	4.2%	(1/24)	3.1%	(7/223)	*P = 0.794*	0.0%	(0/38)	0.2%	(1/432)	*n.a.*
OC43	4.2%	(1/24)	2.2%	(5/223)	*p = 0.573*	0.0%	(0/38)	1.9%	(8/432)	*n.a.*
KU-1	4.2%	(1/24)	0.0%	(0/223)	*n.a.*	2.6%	(1/38)	0.5%	(2/432)	*n.a.*
PIV1	0.0%	(1/24)	0.0%	(0/223)	*n.a.*	5.3%	(2/38)	3.0%	(13/432)	*p = 0.424*
CoV-2	0.0%	(0/49)	1.8%	(4/223)	*n.a.*	10.3%	(6/58)	9.7%	(42/432)	*p = 0.832*

RV/EV: rhinovirus/enterovirus, RSV: respiratory syncytial virus, hMPV: human metapneumovirus, PIV3: parainfluenza virus 3, AdV: adenovirus. NL63: coronavirus NL63, OC43: coronavirus OC43, HKU1: coronavirus HKU1, PIV1: parainfluenza virus 1, CoV-2: severe acute respiratory syndrome coronavirus-2. The whole number in the parenthesis of FC means the patient number who received FA test. *p*-value was calculated by Fisher’s exact test. n.a.: not applicable.

**Table 3:** Comparison of the age, length of hospitalization, type of convulsion, and frequency of first febrile convulsions episode in patients positive or negative for Filmarray^®^ respiratory panel (FA) test, and positive for each virus.

	n	Age (Months)	Hospital Stay (Day)	First Episode	Status Epilepticus	Cluster Spasms
RV/EV	24	30.2 ± 16.8	4.3 ± 2.3	18	21	3
RSV	8	25.7 ± 8.6	4.8 ± 1.7	5	6	2
PIV3	7	24.8 ± 8.1	4.2 ± 0.8	2	5	2
CoV-2	6	45.0 ± 47.9	4.2 ± 1.7	5	2	4
FA (+)	42	27.6 ± 14.5	4.4 ± 2.1	30	28	14
FA (−)	20	40.2 ± 25.9	4.0 ± 1.5	12	14	6

RV/EV: rhinovirus/enterovirus, RSV: respiratory syncytial virus. PIV3: parainfluenza virus 3, CoV-2: severe acute respiratory syndrome coronavirus-2.

## Data Availability

Data are available upon request because of privacy/ethical restrictions.

## Supplementary Materials

The following supporting information can be downloaded at https://www.mdpi.com/article/10.3390/pediatric16020034/s1. Table S1: Point-of-care antigen test for viral infections applied to FC patients admitted between 2012 and 2020.

## Permission to Reproduce Material from Other Sourcest

The published public data were cited and dealt with adequately according to the policy and statement of the quote source.
