# Supplementary material for: Impact of the Omicron Strain on Febrile Convulsions Requiring Hospitalization in Children: A Single-Center Observational Study
Source: Pediatr Rep. 2024 May 14;16(2):399–409. doi: 10.3390/pediatric16020034 (PMC11130907; doi:10.3390/pediatric16020034)
Supplement: Supplementary file 1 [file pediatrrep-16-00034-s001.zip › pediatrrep-2900748-supplementary.pptx]

## Slide 1
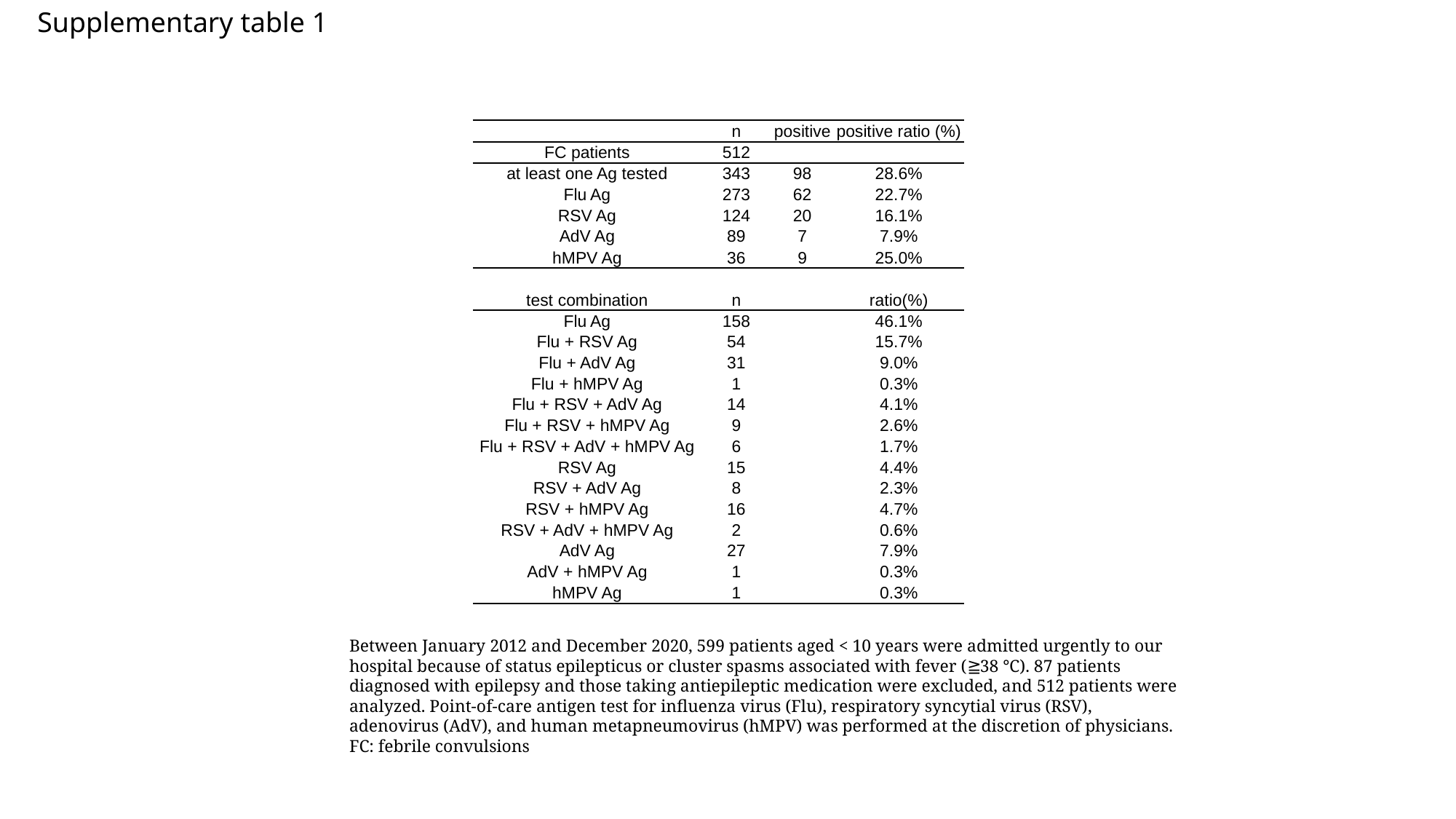

Supplementary table 1
| | | | |
| --- | --- | --- | --- |
| | n | positive | positive ratio (%) |
| FC patients | 512 | | |
| at least one Ag tested | 343 | 98 | 28.6% |
| Flu Ag | 273 | 62 | 22.7% |
| RSV Ag | 124 | 20 | 16.1% |
| AdV Ag | 89 | 7 | 7.9% |
| hMPV Ag | 36 | 9 | 25.0% |
| | | | |
| test combination | n | | ratio(%) |
| Flu Ag | 158 | | 46.1% |
| Flu + RSV Ag | 54 | | 15.7% |
| Flu + AdV Ag | 31 | | 9.0% |
| Flu + hMPV Ag | 1 | | 0.3% |
| Flu + RSV + AdV Ag | 14 | | 4.1% |
| Flu + RSV + hMPV Ag | 9 | | 2.6% |
| Flu + RSV + AdV + hMPV Ag | 6 | | 1.7% |
| RSV Ag | 15 | | 4.4% |
| RSV + AdV Ag | 8 | | 2.3% |
| RSV + hMPV Ag | 16 | | 4.7% |
| RSV + AdV + hMPV Ag | 2 | | 0.6% |
| AdV Ag | 27 | | 7.9% |
| AdV + hMPV Ag | 1 | | 0.3% |
| hMPV Ag | 1 | | 0.3% |
| | | | |
Between January 2012 and December 2020, 599 patients aged < 10 years were admitted urgently to our hospital because of status epilepticus or cluster spasms associated with fever (≧38 ℃). 87 patients diagnosed with epilepsy and those taking antiepileptic medication were excluded, and 512 patients were analyzed. Point-of-care antigen test for influenza virus (Flu), respiratory syncytial virus (RSV), adenovirus (AdV), and human metapneumovirus (hMPV) was performed at the discretion of physicians. FC: febrile convulsions
